# Volcanic Ash and the Respiratory Immune System - Possible Mechanisms behind Reported Infections

**DOI:** 10.1289/ehp.121-a197

**Published:** 2013-06-01

**Authors:** Bob Weinhold

**Affiliations:** Bob Weinhold, MA, has covered environmental health issues for numerous outlets since 1996. He is a member of the Society of Environmental Journalists.

Ash plumes erupting from volcanoes can cause a range of health effects following exposure either to the initial emissions or to deposited material that becomes resuspended days, months, or years later. Researchers from the United States, Iceland, and Australia now report in *EHP* evidence that exposure to volcanic ash may both foster bacterial growth in the respiratory system and weaken the ability of the immune system to limit that growth, possibly contributing to respiratory infections that have been documented in relation to past eruptions.^1^

Each year eruptions occur at about 50–70 above-sea locations; eruptions have been documented for about 550 volcanoes in all, and roughly 1,300 have been active at some point in the past 10,000 years.^2^ Some of this activity generates ash plumes that affect potentially millions of people. Volcanic ash can contain significant quantities of respirable particles of rock, minerals, and volcanic glass, and eruptions also emit hazardous gases such as sulfur dioxide, hydrogen sulfide, hydrochloric acid, and hydrofluoric acid.^3^

The 20 March 2010 eruption of the Icelandic volcano Eyjafjallajökull (pronounced, roughly, AY-yah-FYET-lah-YOH-kuhl), another larger eruption on 14 April 2010, and ongoing volcanic activity through late May 2011 ejected numerous major ash plumes that spread far and wide. For the current study, the researchers primarily used one sample of this ash that was collected on the ground 15 April 2010 at a location 58 km from the volcano. The ash was composed of a complex mixture of aluminosilicate volcanic glass and minerals including iron, titanium, manganese, zinc, strontium, and barium. The researchers used a ubiquitous and potent opportunistic human bacterium, *Pseudomonas aeruginosa*,^4^ to test for possible interaction between ash and this sample infectious agent in primary human airway epithelial cells, primary rat alveolar epithelial cells, and primary human and rat alveolar macrophages.

**Figure:**
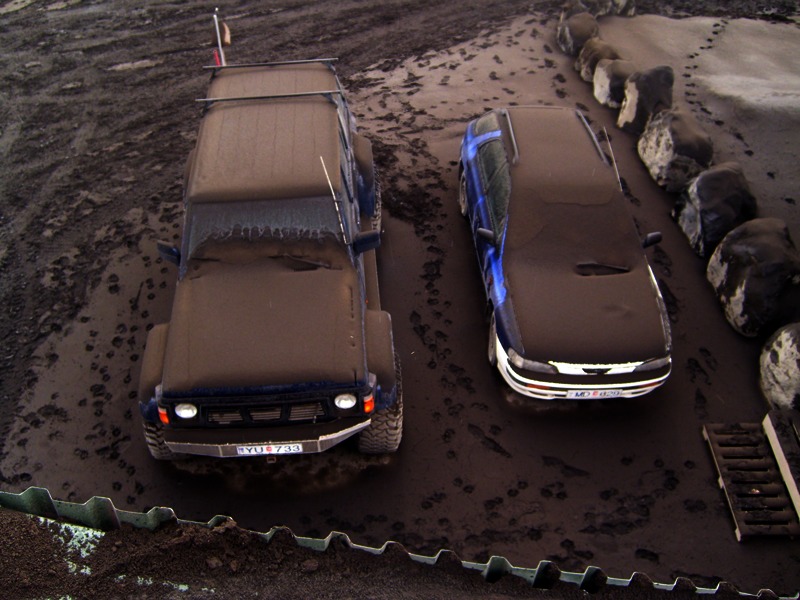
Iron-rich ash from the Eyjafjallajökull eruption spread over thousands of square kilometers. © Jon Snorrason/EPA/Corbis

**Figure:**
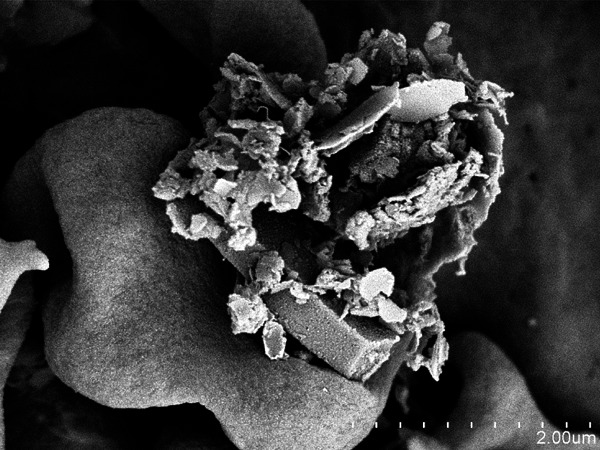
A high-power scanning electron microscopy image shows a smooth-surfaced alveolar macrophage in the process of engulfing (“phagocytizing”) ash particles collected by the authors. Martha M. Monick/University of Iowa

The authors conclude that reports of increased respiratory infections following eruptions may reflect an ability of volcanic ash to spur the growth of bacteria as well as interfere with innate immunity. They found a statistically significant increase in growth of the cultured bacteria after ash exposure, possibly due in part to bioavailable iron in the ash. They treated the cultured bacteria with antimicrobial peptides, some with and some without ash included. Bacterial growth was inhibited by the peptides as expected, but less so when ash was included. There was some evidence that ash exposure might impede functions of macrophages including autophagy, bacterial killing ability, and inflammatory responses, but those results didn’t reach statistical significance. However, unlike particles found in cigarette smoke and diesel exhaust, the ash did not kill human airway epithelial cells or rat alveolar epithelial cells nor did it disrupt epithelial cell barrier integrity.^1^

The study results are “very significant, given there are no current risk assessments or therapeutic interventions in place for avoiding potential respiratory infections following exposure to ash, especially for sensitive groups such as asthmatics, infants, and the elderly,” says Kelly BéruBé, director of the Lung and Particle Research Group at Cardiff University in Wales.

But BéruBé cautions that numerous limitations of the study make it premature to apply the findings to real-world human exposures. For instance, the ash sample tested was a mixture of coarse and fine particles collected on the ground rather than from the air at a height typical of inhalation. She says this makes it impossible to determine if the effects were caused by the fine particles in this mixture (a size that has been identified in a wealth of studies as especially harmful). In addition, the cultured human cells tested came from only two people and thus aren’t representative of many important variables such as age, health status, risk behaviors, and area of geographic residence. The study also did not address any possible confounding effects of coexposure to toxic gases emitted during eruptions.

Volcanic ash can vary substantially depending on factors such as the geochemistry of the individual volcano, the phase of and distance from the eruption, and the degree of posteruption weathering the ash has undergone. Even for the one sample in this study, the published information was too limited to allow accurate analysis of all its mineralogical characteristics, making the study results “problematic for comparisons with other volcano ash,” says Tim Jones, a senior lecturer in environmental geology at Cardiff University. Nonetheless, he says, “This is certainly a step forward in our understanding of the bioreactivity of volcanic ash.”
